# Validation of Wearable Device Consisting of a Smart Shirt with Built-In Bioelectrodes and a Wireless Transmitter for Heart Rate Monitoring in Light to Moderate Physical Work

**DOI:** 10.3390/s22239241

**Published:** 2022-11-28

**Authors:** Yuki Hashimoto, Rieko Sato, Kazuhiko Takagahara, Takako Ishihara, Kento Watanabe, Hiroyoshi Togo

**Affiliations:** NTT Device Innovation Center, NTT Corporation, 3-1, Atsugi 243-0198, Japan

**Keywords:** heart rate, wearable device, holter monitor, physical load

## Abstract

Real-time monitoring of heart rate is useful for monitoring workers. Wearable heart rate monitors worn on the upper body are less susceptible to artefacts caused by arm and wrist movements than popular wristband-type sensors using the photoplethysmography method. Therefore, they are considered suitable for stable and accurate measurement for various movements. In this study, we conducted an experiment to verify the accuracy of our developed and commercially available wearable heart rate monitor consisting of a smart shirt with bioelectrodes and a transmitter, assuming a real-world work environment with physical loads. An exercise protocol was designed to light to moderate intensity according to international standards because no standard exercise protocol for the validation simulating these works has been reported. This protocol includes worker-specific movements such as applying external vibration and lifting and lowering loads. In the experiment, we simultaneously measured the instantaneous heart rate with the above wearable device and a Holter monitor as a reference to evaluate mean absolute percentage error (MAPE). The MAPE was 0.92% or less for all exercise protocols conducted. This value indicates that the accuracy of the wearable device is high enough for use in real-world cases of physical load in light to moderate intensity tasks such as those in our experimental protocol. In addition, the experimental protocol and measurement data devised in this study can be used as a benchmark for other wearable heart rate monitors for use for similar purposes.

## 1. Introduction

The risk of injury associated with the accumulation of physical load is unavoidable in many workplaces. Identifying and correcting these potential risk factors is important for worker safety, health, and productivity [1]. Wearable devices that can monitor the user’s biometric information in real time have been reported to enable quantitative management of the user based on biometric information and to be technologically superior to conventional subjective monitoring methods such as questionnaires [2,3,4,5], which are suitable for monitoring workers. One of the most important pieces of biological information for such monitoring is heart rate. By measuring time series data, it is possible to obtain various indicators derived from the heart rate such as resting heart rate, maximum heart rate, reserve heart rate, exercise intensity, and stress [6,7]. One of the most widespread wearable heart rate monitors for consumers is the wristband-type sensor [8,9]. This sensor measures changes in blood flow in blood vessels (pulse wave) using optical methods such as infrared light, and the mechanism is based on the photoplethysmography (PPG) method. PPG detects the pulse rate, corresponding to heart rate, which can be detected from the R wave on an electrocardiogram (ECG). In addition, shirt-type or belt-type and other textile based sensors equipped with ECG measurement electrodes that can be placed in close contact with the chest or other parts of the surface body are also widespread [10,11,12,13,14,15,16]. For heart rate measurement, PPG-type sensors detect pulse rates from blood flow changes in the vicinity of the measurement area. Consequently, the detected pulse rate does not necessarily match the timing of the R wave in the ECG signal due to the influence of blood flow changes, resulting in an error of 20 ms or more in heart rate detection compared to ECG type sensors [17,18]. In addition, motion artifacts can be introduced during body movement, making the peak itself undetectable. Thus, ECG-type sensors are superior to PPG-type sensors in terms of the accuracy of heart rate monitoring [18].

Taking the advantage of ECG type sensors into account, our previous studies have developed and investigated a wearable device consisting of a smart textile for measuring ECG signal on the body surface and a transmitter for analyzing ECG signal and wireless transmission to an external device [10,19,20]. In the next step, we are planning to apply the wearable device to worker monitoring under specific work such as maintenance work for the network system, which is one of the core business activities in telecommunication companies such as our group company. However, standard exercise protocols simulating such work activities for the validation have not been reported. In this study, we designed a light to moderate intensity exercise protocol that assumes such work for the validation of the wearable device was conducted with this protocol. Specifically, we conducted simultaneous measurement with the device and a Holter monitor, known as the gold standard device in the medical field, under the developed protocol and compared the results.

## 2. Materials and Methods

### 2.1. Device

#### 2.1.1. Developed Wearable Device

**Figure 1:**
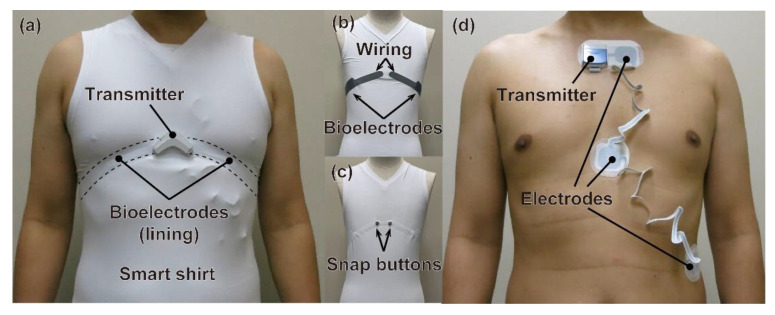
(**a**) Wearing the developed wearable device. (**b**) Bioelectrodes and wiring sewn into the lining of the smart shirt. (**c**) Snap buttons on the front side of the smart shirt. (**d**) Wearing the reference Holter monitor.

Figure 1a shows an overview of the developed wearable device. The device consists of a smart shirt (C3fit IN-pulse, GOLDWIN Inc., Tokyo, Japan) with bioelectrodes and a transmitter (Tx02, NTT TechnoCross Corporation, Tokyo, Japan) with the functions of biosignal analysis and wireless transmission. hitoe^TM^, sewn into the lining of the shirt as the bioelectrodes, adheres closely to the skin and enables stable data acquisition even during exercise (Figure 1b) [21]. The performance fabric hitoe^TM^ is a fiber material jointly developed by Toray Industries, Inc. and Nippon Telegraph and Telephone Corporation, and is the registered trademark of the two companies. The transmitter is relatively light (12 g) compared to that in other commercially available wearable heart rate sensors. It can be connected to the bioelectrodes on the shirt through snap buttons (Figure 1c), and its heartbeat detection algorithm can analyze the heart rate from single-lead ECG signals at 1 kHz obtained through the bioelectrodes [19]. The detection algorithm is implemented with firmware installed on the wearable device has mainly two steps. The peak of the inverted time difference in the ECG is detected, and whether the peak is derived from an R wave or not is determined by checking whether the peak has enough time width corresponding to the QRS complex before and after the peak. The algorithm enables higher recall to ECG waveforms that include noise caused by body motion than a conventional detection algorithm based on a threshold-based peak search [19]. It can also measure temperature, humidity, physical activity levels, numbers of steps, and posture using internal sensors. In addition, a wireless communications circuit is implemented to transmit acquired data to external devices such as smartphones, and the data can be monitored via a dedicated application on the external device [10].

#### 2.1.2. Reference Holter Monitor

Figure 1d shows an overview of the medical Holter ECG device (Cardy 305 pico, SUZUKEN Co., Ltd., Nagoya, Japan) used to obtain the reference heart rates [22]. The device consists of a transmitter and three disposable gel electrodes. The device is capable of 3-lead medical grade ECG measurement at 125 Hz by bonding the electrodes to the skin. In addition, the heartbeat detection algorithm can calculate time-series data of the heart rate from the ECG data. In the experiment, heart rates were calculated from CM5 lead ECG with one electrode placed near the neck and the other near the armpit. For simultaneous measurement by the wearable device and Holter monitor, one of the two electrodes of the Holter monitor was shifted from its standard position in the CM5 lead to avoid interference between them. We confirm with the manufacturer of the Holter monitor that the operation has no problems in terms of ECG measurement and heart rate detection.

### 2.2. Experimental Conditions

#### 2.2.1. Subjects and Ethical Approval

Eight healthy men with a mean (±SD) age of 38.8 ± 8.5 years, height of 179.5 ± 5.0 cm, and weight of 66.3 ± 5.8 kg voluntarily participated in the experiment. Each subject provided written, informed consent after all potential risks and procedures were explained. All experimental procedures and protocols conformed to the Declaration of Helsinki and were approved by the review boards in the Japanese Society for Wellbeing Science and Assistive Technology. None of the subjects were taking medications that might affect cardiopulmonary function during the experiment.

#### 2.2.2. Experimental Design

**Figure 2:**
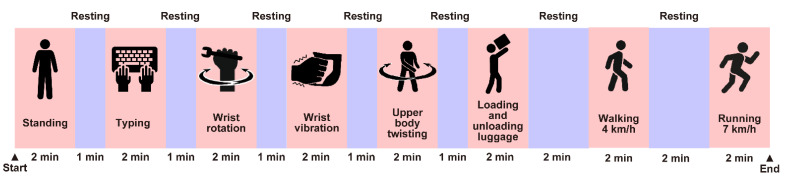
Timeline of the experiment in each subject. The protocol consists of the eight exercise protocols (standing, typing, wrist rotation, wrist vibration, upper body twisting, loading and unloading luggage, walking at 4 km/h, and running at 7 km/h) with a break between each protocol.

**Figure 3:**
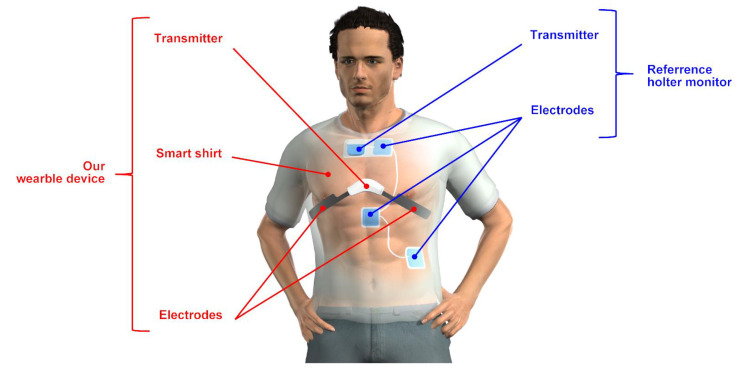
Illustration of the simultaneous heart rate measurement with the wearable device and Holter monitor. The electrodes and transmitter of the Holter monitor are inside the smart shirt and bonded to the skin. The electrodes on the smart shirt are in close contact with the skin due to the compression of the shirt.

Figure 2 shows an overview of the designed experimental protocol, which consists of eight different exercises: standing, typing, wrist rotation, wrist vibration, upper body twisting, loading and unloading luggage, walking at 4 km/h, and running at 7 km/h, simulating the role of a network system maintenance tasks. In accordance with international standards (ISO 8996:2021), all exercises are classified as light to moderate intensity except running at 7 km/h [23]. Note that these movements are not entirely original to network system maintenance work, but are a combination of several types of work and movements. Typing is the action of sitting in a desk chair and typing on a keyboard on the desk. Wrist rotation is an action of sitting on a desk chair and rotating both wrists alternately clockwise and counterclockwise at a speed of 90 rotations per minute while the arms are bent at right angles and fixed to the table. Wrist vibration is an action of holding a device (Swingbeat, YA-MAN CO., Ltd., Tokyo, Japan) that can apply vibration to both wrists at 13 Hz in a standing position. This condition assumes a situation in which a worker is operating an electric tool with vibration. Upper body twisting represents a standing upper body twisting movement at a speed of 60 rotations per minute, alternating clockwise and counterclockwise. Loading and unloading luggage represents placing luggage of 400 mm × 150 mm × 250 mm in size and 1 kg in weight on a shelf at a height of 2 m and removing the luggage from the shelf and placing it on the ground, repeatedly at a period of four seconds. This operation aims to assess measurement errors due to misalignment between the bioelectrodes and skin and due to superimposed myoelectricity caused by vertical arm movements. Walking and running were performed on a treadmill (T5000, Johnson Health Tech Japan Co., Ltd., Tokyo, Japan). Each exercise was set to last two minutes. One-minute resting time between each exercise was set until the loading and unloading of the luggage. Two-minute resting time between each exercise was set after that. The measurement data from the last minute of the second half of each exercise was used for the analysis. During the experiment, the subjects wore the wearable device and the reference Holter monitor as shown in Figure 3 to simultaneously obtain ECG time series data and detected heart rates from each device. Each subject’s shirt size was selected based on his physique and the device specifications. The experiment was conducted in our laboratory room during the summer of 2021. The room temperature was 28 °C, and the humidity was between 68% and 78% RH. These environmental conditions are considered adequate to simulate the outdoor summer environment in Japan where it is difficult to control temperature and humidity.

### 2.3. Data Analysis

An evaluation was performed to determine if the heart rate acquired by the wearable device is valid for each action in the experiment compared to that of the Holter monitor. Time synchronization of heart rate data was performed based on the time stamps associated with the heart rate data acquired from each device. The heart rates that were determined to be invalid by the original algorithm in the Holter monitor and the heart rates acquired on the wearable device corresponding to them were excluded from the analysis. The heart rate data acquired with the wearable device after the above processing included heart rate data resulting from false detection of the R wave and missing R waves due to body movement, which, along with such data on the Holter monitor, were also excluded. The excluded data were separately calculated as indices of the false detection rate and missing rate of heart rate in the wearable device. For the data set that remained after the above data exclusion processes, the mean absolute percentage error (MAPE) of the heart rate acquired by the wearable device relative to that of the Holter monitor for each movement was calculated by Equation (1) and compared with a threshold value (MAPE = 5%).

(1) $$\mathrm{MAPE}\;\left(\%\right)=\frac{100}{n}\overset{n}{\underset{i=1}{\sum }}\left|\frac{x_{i}-x_{ref,\;i}}{x_{ref,\;i}}\right|$$

where *x_i_* [bpm] is heart rate measured by the wearable device after the above data exclusion process, *x_ref,i_* [bpm] is heart rate measured by the Holter monitor corresponding to *x_i_*, and *n* is total number of heart beats per minute. The threshold value was set based on the advice of experts in medical and thermal physiology to be considered equivalent to the reference data to evaluate the validity of the heart rate acquired by the wearable device for each movement. The validity was ascertained by an equivalence test with α of 0.05, β of 0.20, the standardized effect size of 33.9, where we compared the 95% confidence interval calculated from the measured MAPE with the threshold value. The number of subjects was sufficient for the validation.

## 3. Results and Discussion

**Figure 4:**
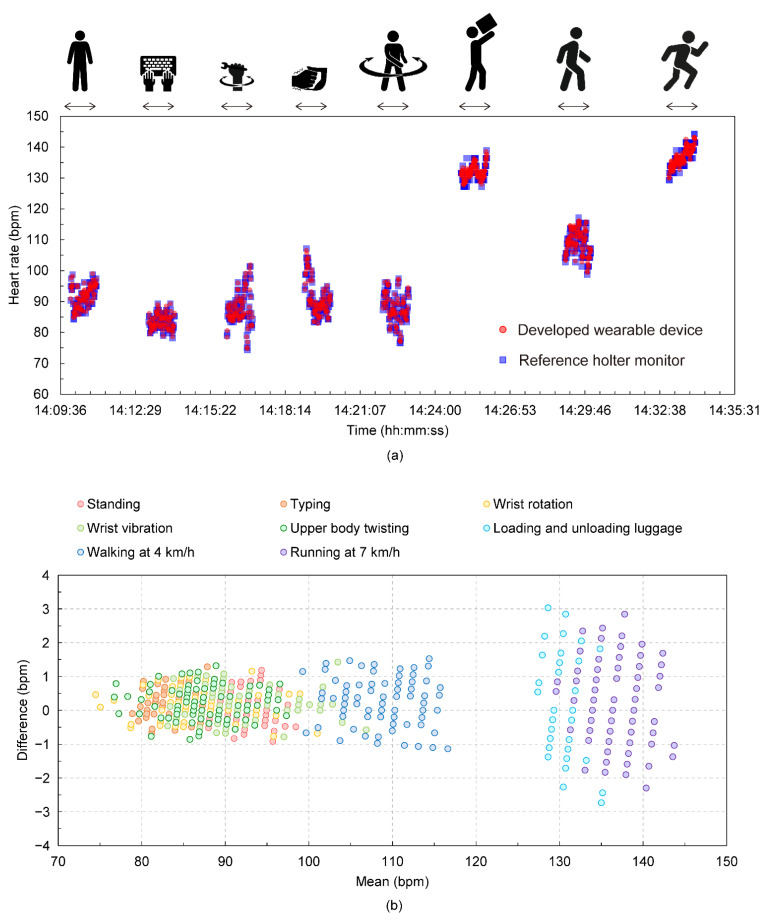
(**a**) Example of time series of heart rate data obtained from the wearable device and Holter monitor at each movement. (**b**) Bland–Altman plots comparing the heart rates in the wearable device and Holter monitor during entire protocol including all measurement in Figure 4a.

An example of heart rate measurements is shown in Figure 4. Figure 4a shows its time series data. The figure shows that heart rate data in the previous movement did not affect that of the current movement, which indicates that the designed resting intervals between the movements and timing of measurement for each movement are appropriate. Figure 4b shows the Bland–Altman plots comparing the heart rates in the wearable device and Holter monitor during entire protocol including all measurement in Figure 4a. Standard deviations in heart rate differences for each movement are 0.50 bpm (Standing), 0.44 bpm (Typing), 0.43 bpm (Wrist rotation), 0.49 (Wrist vibration), 0.47 bpm (Upper body twisting), 1.10 bpm (Loading and unloading luggage), 0.62 bpm (Walking at 4 km/h), and 1.07 (Running at 7 km/h), respectively. Averaged mean heart rates for each movement are 91.6 bpm (Standing), 83.4 bpm (Typing), 87.4 bpm (Wrist rotation), 91.1 (Wrist vibration), 87.7 bpm (Upper body twisting), 131.7 bpm (Loading and unloading luggage), 109.9 bpm (Walking at 4 km/h), and 135.4 (Running at 7 km/h), respectively. Compared to the first five movements and the condition of walking at 4 km/h, there appears to be a slight difference in the heart rates acquired by the Holter monitor and the wearable device in other movements, and the differences are tended to be large with increasing the heart rate.

**Figure 5:**
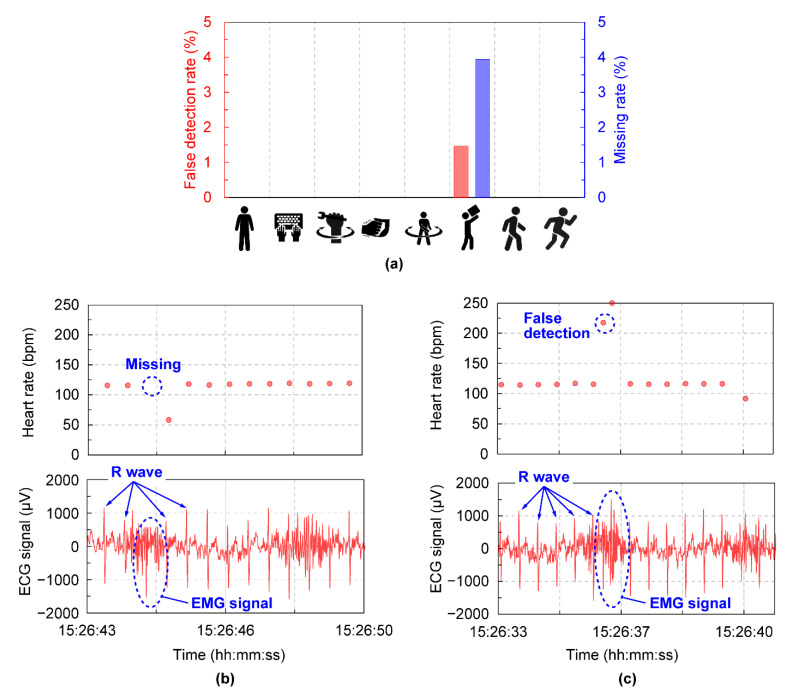
(**a**) False detection rate and missing rate of heart rate in the wearable devices in each motion. This data includes the subject’s measurement data described in Figure 4. (**b**) A typical example of missing heart rate data due to missed R wave in ECG signal measured by the wearable device. (**c**) A typical example of calculation of abnormal heart rate due to false detection of R wave in ECG signal measured by the wearable device.

Figure 5a shows the false detection rate and missing rate of the heart rate acquired by the wearable device. Both cases occurred only during the loading and unloading of luggage, at 1.47% and 3.95%, respectively. This is due to the superimposition of myopotential components on the ECG signal during that motion, and this phenomenon was not detected for all subjects; it was due to individual differences. Figure 5b and c show examples of ECG signals and corresponding heart rate acquisitions during the missing and false detection. These results confirm the superimposition of myoelectric components caused by the lifting and lowering of luggage on the ECG signals. It is considered that whether the missing or false detection occurred depended on the timing of myoelectric components and R wave appearance. This suggests that missing or false heart rate detection by this wearable device may occur during certain operations, but the percentage is low, and we confirmed that the device was able to measure heart rates in many operations with almost no missing or false detections.

**Figure 6:**
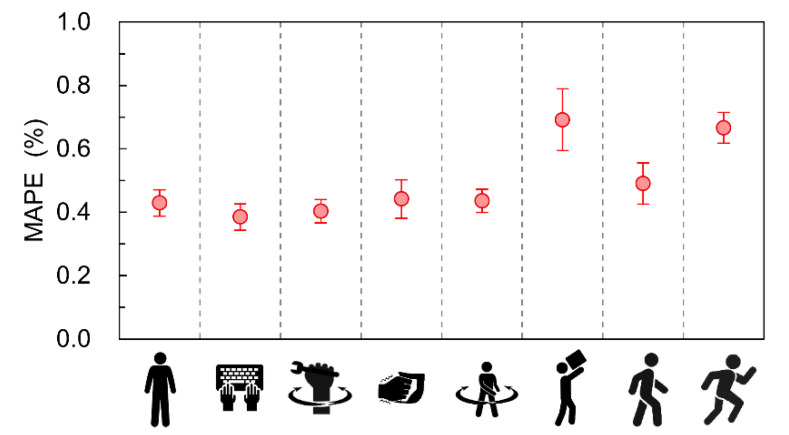
MAPE value of the heart rate acquired by the wearable device relative to the heart rate acquired by the Holter monitor for each movement. The data are shown as mean ±the 95% confidence interval. This data includes the subject’s measurement data described in Figure 4.

**Figure 7:**
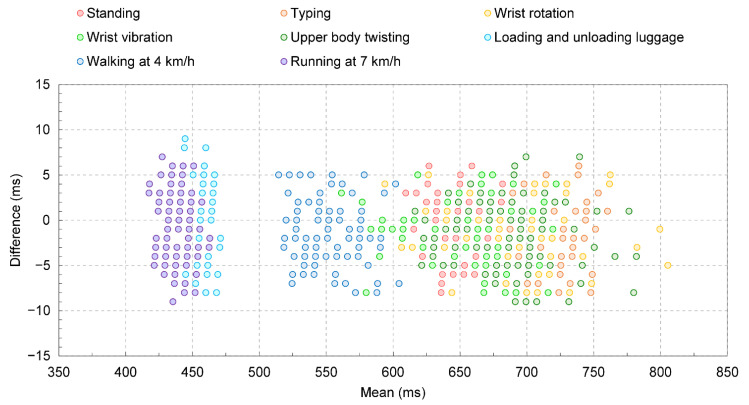
Bland–Altman plots comparing the RR intervals in the wearable device and Holter monitor during entire protocol including all measurement in Figure 4a.

Figure 6 shows the MAPE calculations for the heart rate acquired by the wearable device relative to the heart rate acquired by the Holter monitor for each movement. Statistically, similar to the trend confirmed by the individual data as shown in Figure 4, the first five movements had smaller errors than the last three movements. Based on the results in Figure 4, the higher the heart rate was during operation, the higher the MAPE value. One error factor is the effect of the sampling intervals during the measurement. Figure 7 shows the Bland–Altman plots comparing the RR intervals in the wearable device and Holter monitor during entire protocol including all measurement in Figure 4a. According to the figure, it is observed that the error in the RR interval is distributed in the range of −9 ms to 9 ms. The sampling intervals of the wearable device and the Holter monitor are 1 ms and 8 ms, respectively, which can result in errors in the RR interval of up to 9 ms if it were not for the effect of the heartrate detection algorithms. This result is considered to be due to the effect of the sampling intervals in the devices. For example, in the case of standing, the average heart rate during the operation was about 91.6 bpm, as shown in Figure 4, so the sampling interval’s effect can result in the MAPE of up to 1.4%. In the case of loading and unloading luggage, the average heart rate during the operation was about 131.7 bpm, which can result in the MAPE of up to 2.0% due to the effect of the sampling intervals. Comparing the above discussion with the result in Figure 6, one sees that the error ratio between “standing” and “loading and unloading luggage” estimated from the sampling rate is about the same as the ratio of the MAPE values in Figure 6. Therefore, it is considered that the influence of factors other than the sampling intervals, such as the heart rate extraction algorithm, is relatively small. The obtained index values of MAPE and total heartbeats per minute are summarized in Table 1. These values are calculated based on measurement data from the eight subjects. The table shows that the MAPE was 0.92% or less for all exercise protocols conducted.

Throughout the entire experiment, the MAPE values were smaller than the threshold value (5%). This result indicates that the obtained heart rates by the wearable device are equivalent to the reference Holter monitor within the range of the movements performed in the experiment. The above results support the validity of the heart rate obtained by the wearable device under the operating protocol.

## 4. Conclusions

The purpose of this study is to evaluate the validity of the heart rate measured by our developed wearable device under the exercise protocol that simulates actual works, with a view to utilizing the wearable device for monitoring workers at actual work sites. For the validation, we developed a new exercise protocol that simulates light to moderate physical works such as the maintenance works of the network system that we intend to utilize. Under the protocol, we compared the heart rates measured by the wearable device and the reference Holter monitor attached to the subjects.

As a result of the validation experiment, it was confirmed that the heart rate could be measured without false detections and losses in the R wave in most cases during the entire exercise protocol, although 1.47% of the R wave was falsely detected and 3.95% of the R wave was missed during loading and unloading luggage. We confirmed that the heart rate could be obtained from the wearable device with an error of less than 0.92% in MAPE compared to that of the Holter monitor in all exercise protocols. This value is well below the measurement error threshold of the heart rate required for monitoring workers, which was set in accordance with the advice of experts, and statistically indicates that the heart rate obtained from the wearable device under this protocol is reasonable.

We consider that these results not only demonstrate the feasibility of our wearable device in actual work settings, but also can be used as a benchmark for future validations of wearable heart rate monitors for similar purposes.

## Figures and Tables

**Table 1:** Average of mean absolute percentage error (MAPE), maximum MAPE and total beats per minutes for eight subjects in each protocol. Average of MAPEs are expressed as mean ±95% confidence interval (CI).

Protocol	Average MAPE ± 95%CI (%)	Maximum MAPE (%)	Total Beats per Minute
Standing	0.43 ± 0.045	0.48	84
Typing	0.39 ± 0.050	0.46	73
Wrist rotation	0.40 ± 0.044	0.50	77
Wrist vibration	0.44 ± 0.073	0.58	84
Upper body twisting	0.44 ± 0.044	0.50	80
Loading and unloading luggage	0.69 ± 0.116	0.92	110
Walking at 4 km/h	0.49 ± 0.078	0.64	98
Running at 7 km/h	0.67 ± 0.058	0.78	130

## Data Availability

Data sharing not applicable.
